# Evolution of complete proteomes: guanine-cytosine pressure, phylogeny and environmental influences blend the proteomic architecture

**DOI:** 10.1186/1471-2148-13-219

**Published:** 2013-10-03

**Authors:** Wanping Chen, Yanchun Shao, Fusheng Chen

**Affiliations:** 1 Key Laboratory of Environment Correlative Dietology, Huazhong Agricultural University, Wuhan, Hubei Province 430070, China; 2 National Key Laboratory of Agro-Microbiology, Huazhong Agricultural University, Wuhan, Hubei Province 430070, China; 3 College of Food Science and Technology, Huazhong Agricultural University, Wuhan, Hubei Province 430070, China

**Keywords:** Proteome, Evolution, GC pressure, Phylogeny, Environmental influence

## Abstract

**Background:**

Guanine-cytosine (GC) composition is an important feature of genomes. Likewise, amino acid composition is a distinct, but less valued, feature of proteomes. A major concern is that it is not clear what valuable information can be acquired from amino acid composition data. To address this concern, in-depth analyses of the amino acid composition of the complete proteomes from 63 archaea, 270 bacteria, and 128 eukaryotes were performed.

**Results:**

Principal component analysis of the amino acid matrices showed that the main contributors to proteomic architecture were genomic GC variation, phylogeny, and environmental influences. GC pressure drove positive selection on Ala, Arg, Gly, Pro, Trp, and Val, and adverse selection on Asn, Lys, Ile, Phe, and Tyr. The physico-chemical framework of the complete proteomes withstood GC pressure by frequency complementation of GC-dependent amino acid pairs with similar physico-chemical properties. Gln, His, Ser, and Val were responsible for phylogeny and their constituted components could differentiate archaea, bacteria, and eukaryotes. Environmental niche was also a significant factor in determining proteomic architecture, especially for archaea for which the main amino acids were Cys, Leu, and Thr. In archaea, hyperthermophiles, acidophiles, mesophiles, psychrophiles, and halophiles gathered successively along the environment-based principal component. Concordance between proteomic architecture and the genetic code was also related closely to genomic GC content, phylogeny, and lifestyles.

**Conclusions:**

Large-scale analyses of the complete proteomes of a wide range of organisms suggested that amino acid composition retained the trace of GC variation, phylogeny, and environmental influences during evolution. The findings from this study will help in the development of a global understanding of proteome evolution, and even biological evolution.

## Background

The dramatic increase of genomic data in public databases for a wide variety of species as the result of plummeting costs and newly introduced sequencing technologies, has set off a wave of post-genomic research [1,2]. One major goal of the post-genome analyses is to characterize features such as composition, content, and functionality of genomes and/or proteomes and to decipher their connection to already known biological characteristics [1,3]. Guanine-cytosine (GC) content is a well-known feature of genomes, similarly, amino acid composition is the distinct feature of proteomes [4]. However, the many *in silico* studies on proteomes have focused mainly on the functional annotation of individual proteins in a proteome [5]. Comparatively, the distribution of amino acids on the proteomic scale has received far less attention. Thus, it has been suggested that information about the amino acid compositions of proteomes should be provided in databases, as is now done routinely for the GC content of genomes [6]. Proteomic architecture carries striking signatures of biological traits, and its compositional biases are closely associated with already recognized internal factors, such as GC variation, and emerging external factors, such as species phylogeny and environmental niche [4-7]. Elucidating the associations between proteomic architecture and such factors is essential to achieve a better understanding of the evolution of proteomes and even organisms.

After the seminal studies of Sueoka in 1961 [8], many different studies drew the recurring conclusion that amino acid composition, whether at the protein or proteomic level, was essentially under the influence of GC pressure [3,9-13]. It is neither new nor surprising that the use of amino acids with GC-rich codons such as Ala, Arg, Gly, and Pro will increase with increasing genomic GC content, while the use of amino acids with AT-rich codons such as Asn, Ile, Lys, Phe, and Tyr will decrease [10,12-14]. Therefore, it is believed that mutational pressure on DNA composition is a very powerful and pervasive force in long-term protein evolution [13]. However, what response mechanisms the organisms have to counter the potentially deleterious effects of this nucleotide bias-induced change on protein function is still unclear, mainly because the effect of nucleotide bias on amino acid composition is very large and widespread. One hypothesis was that the resulting amino acid sequence changes are nonrandom because the mutational bias is strongly directional, and are not caused by natural selection acting directly on protein function. Consequently, the evolutionary dynamics of these changes cannot be described in terms of either Darwinian selection or random genetic drift [13]. Another subsequent explanation suggested compensatory changes were made to reduce the impact of amino acid bias on protein structure and function. Previous analyses have shown that amino acid substitutions such as Arg for Lys and Ala for Ser are important for the stability of proteins in some species [15].

Studies of interlinkages between species phylogeny and proteomic architecture have been less conducted, primarily due to the restriction of information capacity for 20-dimensional amino acid data, far enough to fully characterize the species diversity. Nevertheless, several previous works have implied a close connection between species phylogeny and the amino acid composition information of their proteomes. For example, an in-depth comparison of proteomic composition across more than 70 genera from all three domains revealed a clear differentiation of archaea, eubacteria, and eukaryote from each other [16]. An extensive analysis of amino acid usage in 208 proteomes showed a clearcut segregation of eukaryotes from prokaryotes [4]. The phylogenetic groups within 1029 bacterial and archaeal strains, generally, could be discriminated from other groups based on the amino acid composition of their proteomes [6]. However, it should be stressed that proteomic amino acid information is limited by capacity, and is capable of discrimination of species only at a general level of phylogenetic classification, such as at the domain level, rather than at a detailed classification level.

It is an emerging view that proteomic amino acid composition is influenced by external factors, namely environmental influences [7]. Throughout evolution, organisms may have used this to increase their fitness to an environmental niche, especially under conditions such as extreme temperature, pressure, pH, and salinity. Regardless of the environmental niche, however, adaptation and maintenance of protein integrity and function seem to be fundamental to the survival of entire organisms [3,5,17-19]. Accordingly, many studies have attempted to relate habitats of organisms to the amino acid composition of their proteins and proteomes. At the habitat level, many studies have investigated the relationship between extreme environments, especially at extremely high temperature and salinity ranges, and the adaptive proteomes to identify amino acid signatures associated with different lifestyles [19-25]. At the phylogeny level, environmental signatures have been studied mostly in the proteomes of unicellular prokaryotes. However, a few studies on higher multicellular eukaryotes have also found that the proteomes of these organisms may be under evolutionary adaptation to their habitats. For example, proteomic comparisons among eleven endothermic and ectothermic vertebrates suggested that the amino acid composition in endothermic vertebrates was biased in the same direction as the composition in the proteomes of thermophilic prokaryotes [26]. Analysis of the proteomes across streptophyte lineages, including charophycean algae and embryophytic plants, showed amino acid compositional shifts during streptophyte transitions to terrestrial habitats [27]. However, previous results from direct comparisons of proteomes associated with different lifestyles can be partly misleading. For example, comparisons among temperature-sensitive species may exaggerate the influence of temperature on amino acid composition and thus mask underlying interactions between environmental niche and proteomic architecture.

The compatibility between the genetic code and amino acid composition of proteomes is also worthy of further study, because it may provide some clues about their co- adaptation. Based only on theoretical probability, amino acid frequency should coincide with the expected random distribution of the 61 sense codons of the universal genetic code. However, the observed frequencies of some amino acids show obvious biases against the expected rates. Most of the deviations can be interpreted as the consequences of selective pressures at the level of nucleotide composition or protein structure and function [7,11]. At the genetic level, as mentioned above, variations in the nucleotide composition could shift amino acid composition noticeably. Moreover, some nucleotide combinations, such as the dinucleotide cystosine-guanine (CG), are suppressed in many genomes, which may lead to suppression of the codons that contain the suppressed nucleotides and hence to an underrepresentation of the corresponding amino acids [11,28-31]. At the protein level, on the one hand, the frequency bias of some amino acids can be attributed to structural constraints; for example, Met is principally the initiation amino acid and thus its presence does not follow a random distribution. On the other hand, some amino acids like Cys and Pro occur at significantly less than the expected rates because of their strong influences on protein tertiary structure; that is, Cys can form disulphide bridges, and Pro can terminate helices and introduce turns [11]. Moreover, other factors such as biosynthetic costs, metabolic efficiency, and elements of amino acids also result in their compositional bias [7,32-35].

Unlike previous studies that compared the proteomes of unicellular prokaryotes with habitat tags, a large number of eukaryotes covering a wide taxonomy range were considered in the present study. First, the complete proteome sequences from 63 archaea, 270 bacteria, and 128 eukaryotes in public databases were surveyed for a comprehensive comparison and analysis of their organization, similarity, uniqueness, and variability at the proteomic level to help understand long-term changes in amino acid compositions, selective constraints, and pressures across the proteomes. Then, a global picture was created to help visualize how the amino acid composition of complete proteomes could have been blended mainly by the joint actions of three forces, namely GC pressure, phylogeny, and environmental influences. This study has provided new insights into how proteomes change over evolutionary time and how they are used as the blueprints for organisms.

## Results

### Frequency distribution of amino acids in archaea, bacteria, and eukaryota

**Figure 1:**
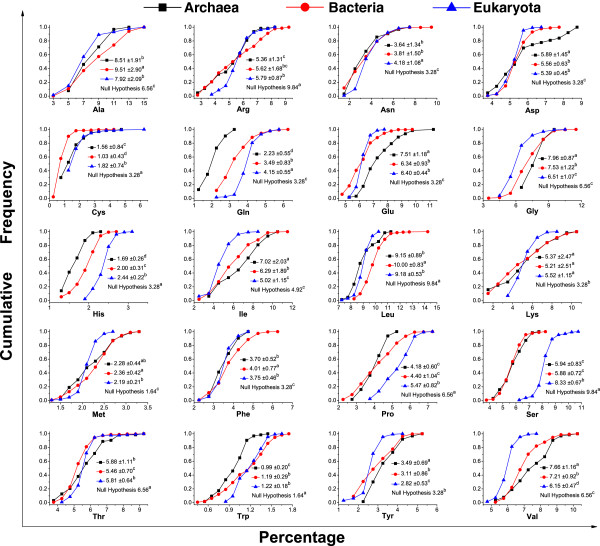
**Cumulative frequency distribution of the 20 standard amino acids in Archaea, Bacteria, and Eukaryota.** Normality tests with SAS statistic software showed that the frequencies of the 20 amino acids in the three domains followed a normal distribution (data not shown). The cumulative frequency ranged from 0 to 1. The distribution followed an S- shaped curve and the largest change in cumulative frequency occurred when the amino acid frequency was highest. The null hypothesis was that there was no difference between amino acid frequency in the proteomes and the frequency based on the universal genetic codes, namely the number of synonymous codons divided by the total number of sense codons. Difference analysis among the amino acid frequencies from different taxa was performed at the 0.05 level using Duncan's multiple-range test. The data were displayed as mean and variance. The superscripts a, b, c and d are Duncan groupings, which indicated that amino acid frequencies within the same grouping were not significantly different. The descending order of the amino acid frequencies correspond to the Duncan groupings from a to d.

The frequency distributions of the 20 standard amino acids were analyzed in Archaea, Bacteria, and Eukaryota (Figure 1). Normality tests indicated that the frequencies of all 20 amino acids in the three domains followed a normal distribution; however, the frequency distributions of the various amino acids differed. Generally, some amino acids such as Ala and Leu made up a large proportion of the overall amino acid composition, while others such as Cys and Trp made up only a small proportion. The dispersion distribution, as reflected by the coefficient of variation (CV), varied by taxa and amino acid. In general, the centralization level of amino acid frequency distribution in taxa by descending order (CV values in ascending order) was from Eukaryota, Bacteria to Archaea. With respect to the different amino acids, some amino acids such as Cys (with CV values over 40%) were in a loose distribution, while other amino acids like Leu (with CV values below 10%) were in a relatively centralized distribution.

The differences and biases in amino acid frequency were studied among the three domains. Translation from the genetic material (DNA or mRNA sequences) to proteins (amino acid sequences) is based on the triplet genetic code [36]; therefore, based merely on probability distribution, the genomic amino acid frequency should be the same as the frequency of the synonymous codons in all the codons in a genome. Therefore, we assumed that there was no difference between the genomic amino acid frequency and the corresponding synonymous codon frequency. The comparison showed differences in the amino acid frequencies among the three domains. The frequencies of Asp, Glu, Gly, Ile, Tyr and Val were higher in Archaea than in Bacteria and Eukaryota, while, the dominant amino acids were Ala, Leu, and Phe in Bacteria, and Asn, Cys, Gln, His, Pro, and Ser in Eukaryota. It is worth mentioning that there seemed to be no frequency difference for Lys among the three domains. We found that the amino acid frequencies differed from the corresponding synonymous codon frequencies in the three domains and two distinguishing groups were found. One group, the “up group”, contained Ala, Asn, Asp, Glu, Lys, Met, and Phe because the frequencies of the amino acids in this group were significantly higher than their assumed frequencies based on the synonymous codon frequencies. The other group, the “down group”, included Arg, Cys, His, Pro, Ser, Thr, and Trp because the frequencies of the amino acids in this group were significantly lower than their assumed frequencies based on the synonymous codon frequencies.

**Figure 2:**
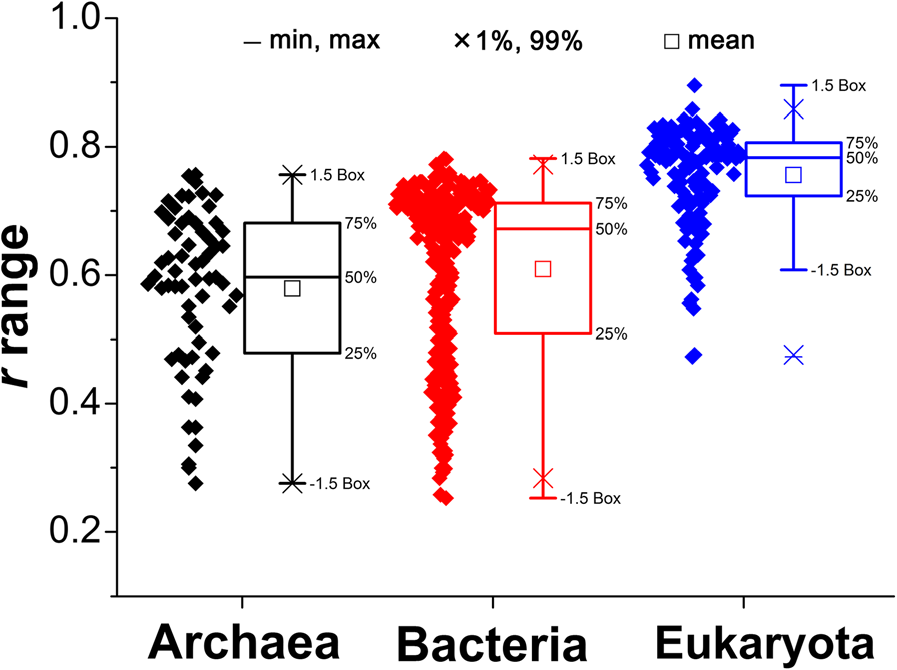
**Correlation between the 20 amino acid frequencies and their corresponding synonymous codon frequencies.** The Pearson correlation coefficient (*r*) was used to evaluate the correlation. Statistical results are shown as data (left) and boxes (right).

The correlation between the overall frequencies of the 20 amino acids and their corresponding synonymous codons was evaluated using Pearson correlation coefficients (Figure 2). We found that the amino acid frequencies in the three domains were all positively and mostly significantly correlated with their corresponding synonymous codon frequencies. This finding suggested that the overall distribution of the 20 amino acids might be consistent with the overall distribution of the synonymous codons, even if individual amino acid frequencies showed differences to the frequencies of their synonymous codon. Moreover, the boxplot of correlation coefficients in the three domains showed that, in Eukaryota, there was a significantly higher correlation between the amino acid frequency and the synonymous codon frequency compared with the correlation in the other two domains (Figure 2).

### Physico-chemical profile of amino acids in archaea, bacteria, and eukaryota

**Figure 3:**
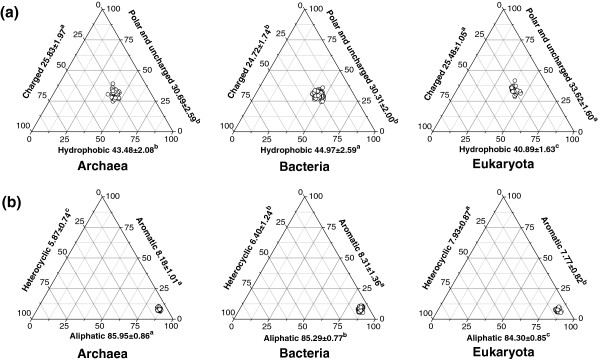
**Physico-chemical framework of the amino acids in the proteomes of Archaea, Bacteria, and Eukaryota. (a)** The 20 amino acids were classified into hydrophobic, charged, and polar and uncharged amino acids based on the polarity of their side chains. **(b)** The 20 amino acids were classified into aliphatic, aromatic, and heterocyclic amino acids based on the chemical structure of their side chains. The related data processing methods were as described in the legend to Figure 1.

A general classification for amino acids is based on the polarity of the side chains; namely, polar and uncharged, charged, and hydrophobic. Among these, the hydrophobic amino acids made up the largest proportion of amino acids in all three domains (over 40%), while the polar and uncharged amino acids (over 30%), and charged amino acids (about 25%) accounted for the rest (Figure 3a). The distribution of the three types of amino acids was relatively centralized (CV values below 8.5%). Archaea, Bacteria, and Eukaryota possessed the highest frequency of charged, hydrophobic, and polar and uncharged amino acids, respectively.

Based on the chemical structure of the side chains, the amino acids can be classified into aliphatic, aromatic, and heterocyclic amino acids. Our analysis showed that the aliphatic amino acids made up the largest proportion of the amino acids in all three domains (about 85%), although, of the three, Archaea had the highest proportion (Figure 3b). Bacteria and Eukaryota had the highest frequencies of aromatic and heterocyclic amino acids, respectively.

### Interlinkage of amino acid frequencies in the tested proteomes

When the correlations between the frequencies of the 20 amino acid were studied, we identified two distinct amino acid groups; one that contained Ala, Arg, Gly, Pro, Trp, and Val, and another that included Asn, Lys, Ile, Phe, and Tyr. The frequencies of the amino acids were positively correlated within each group, but negatively correlated between the two groups (Additional file 1). These associations were consistent in all three domains.

### Principal components of amino acid frequency matrix from the tested proteomes

Principal components of the amino acid frequency in the 20-dimensional matrices for the 461 tested species were analyzed, and the top three components, which together accounted for 71.81% of the total inertia, were listed (Additional file 2). The data showed that principal component 1 (Prin1), which made up the largest proportion, was positively and closely related to the frequencies of Ala, Arg, Gly, Pro, Trp, and Val, but negatively related to the frequencies of Asn, Ile, Lys, Phe, and Tyr. These amino acids had already been found to be correlated as mentioned in the previous section. Therefore, Prin1 was focused mainly on changes among the 11 frequency-dependent amino acids. On the other hand, the second principal component (Prin2) mainly reflected changes among Gln, His, Ser, and Val, and the third principal component (Prin3) mainly focused on changes among Cys, Leu, and Thr.

**Figure 4:**
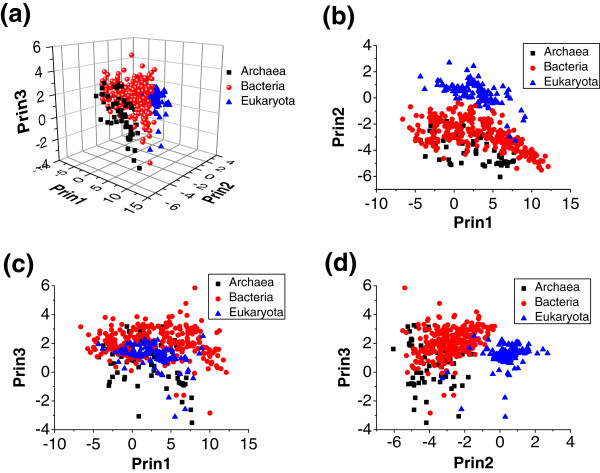
**Principal components analysis of amino acids in the proteomes of species from the three domains.** The distribution of points on the principal factorial planes is shown. One point corresponds to one species. **(a)** Projections onto a 3D principal plane spanned by the principal component 1 (Prin1), Prin2, and Prin3 axes. **(b–d)** Projections onto the three 2D factorial planes in the Prin1, Prin2, and Prin3 axes, respectively.

We compared the distribution of the amino acids in the proteomes of the 461 species from the three domains in the principal component planes (Figure 4) and found that species within the same domain tended to gather together in 3D planes (Figure 4a). The distribution of Archaea was separate from that of Eukaryota, indicating that these two domains might be well distinguished by amino acid composition. Bacteria were clustered between Archaea and Eukaryota. It seemed that Bacteria were well separated from Eukaryota, even though there were a few species from each domain for which the distribution overlapped. However, a large part of the distribution of Archaea overlapped with the distribution of Bacteria. Furthermore, the projections onto the 2D planes in the Prin1 and Prin3 axes showed that there were no obvious distribution differences among the three domains (Figure 4b, c and d). However, the distribution of Eukaryota in the Prin2 axis showed significant differences compared with the distribution of the other two domains. The scores for most of the Eukaryota species in Prin2 were greater than 0, while of the scores for the Archaea and Bacteria species were less than 0.

### Cluster analysis of 461 species based on amino acid frequency differences

**Figure 5:**
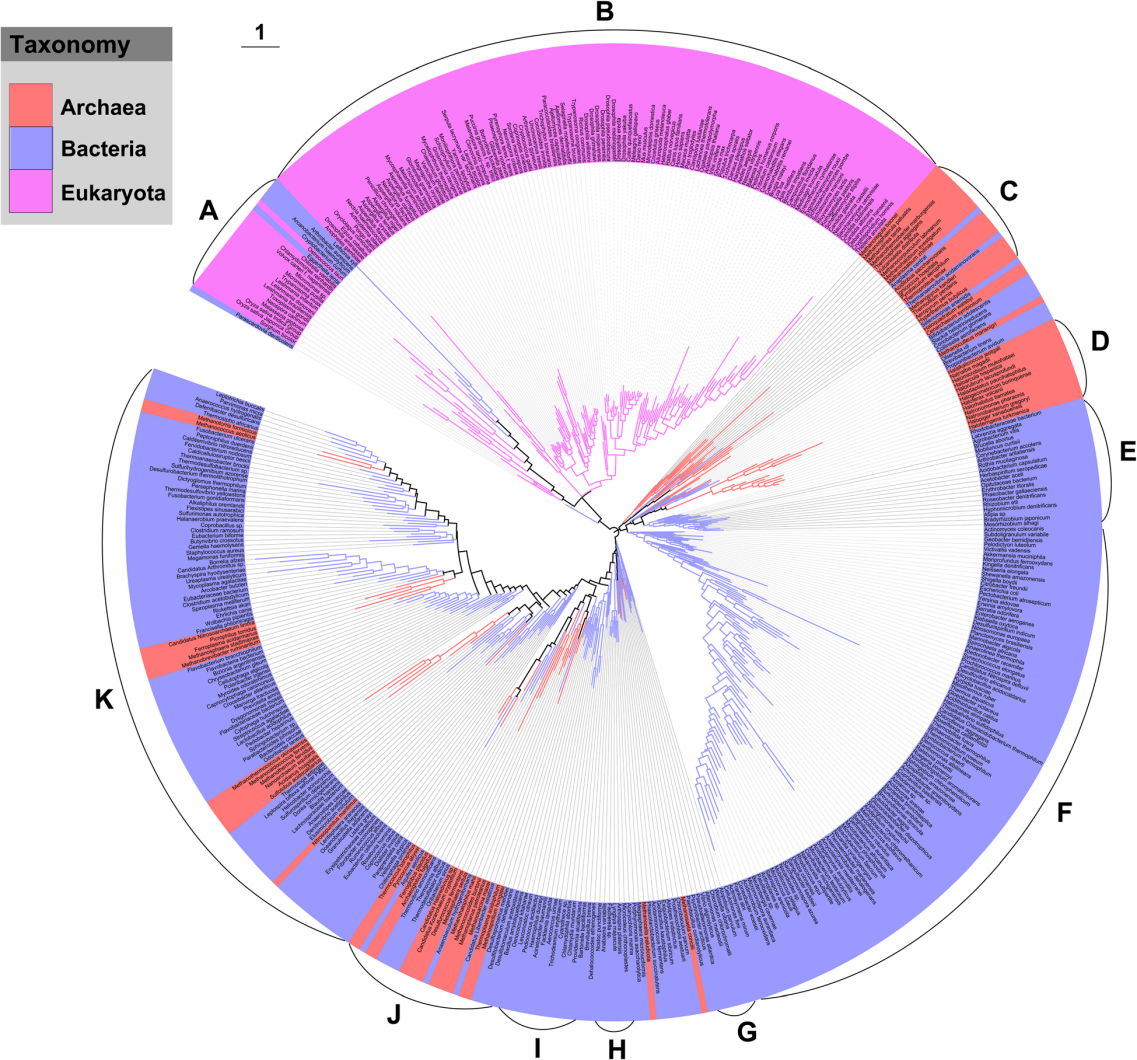
**Clustering tree of 461 species based on amino acid frequency differences.** The inner circle is the phylogenetic tree of 461 species from Archaea, Bacteria, and Eukaryota. The tree was inferred from a Euclidean distance matrix of amino acid frequency with a neighbor-joining method using the Mega 5.05 software and edited on iTOL. Ancestral branches with children that had identical colors were assigned the same color as the children. The outer circle displays the corresponding species, which are covered by different colors to show the taxonomic group as the legend indicated. Each taxon is linked to the corresponding branch by a dotted line.

The species divided into 11 main branches (groups A–K) based on the cluster analysis of the amino acid composition of their proteomes (Figure 5). On the whole, phylogenetically close species were more likely to be clustered together in the tree compared with the more distant species. The Eukaryota species seemed to be distinguished from the other two domains. Although some species from Archaea and Bacteria were clustered in the tree, clear differences in amino acid composition between these two domains were revealed. A taxonomic comparison of the 11 main branches showed that the species in seven of the groups (B, D, E, F, G, H, and I) were from a single domain, while the species in the other four groups were from different domains. As mentioned above, the amino acid distribution in the proteomes of the Bacteria species acted as a transition region because it overlapped with the distribution of either Archaea or Eukaryota species.

The distribution characteristics of the amino acids were compared among the 11 groups (Additional file 3). The group A species were from the Bacteria and Eukaryota domains, and the main members were Actinobacteria, plants, and protists. The proteomes of the species in this group contained the highest frequencies of Ala and Cys, and the lowest frequencies of Phe, Ile, and Tyr, and the aromatic amino acids. Group B species were all Eukaryota, covering all the tested animals and fungi, and some of the plants and protists. The proteomes of the species in this group had the highest frequencies of His, Pro, and Ser, and the lowest frequencies of Gly and Val. The group B proteomes shared a distinctive amino acid framework; for example, the proteomes contained the highest percentages of polar and uncharged amino acids and the lowest percentages of hydrophobic amino acids, and the most heterocyclic amino acids and the least aliphatic amino acids, among the 11 groups. The group C species were mainly Thermoprotei and Methanomicrobia in the Archaea domain. The proteomes of the species in this group had the lowest frequencies of Asp, His, and Gln and no obviously dominant amino acids. The group D species were all Archaea and all were Halobacteria. Interestingly, the Halobacteria proteomes showed a distinct distribution in amino acid frequency with Asp, Glu, Gly, Thr, and Val being the amino acids with highest frequencies and with many other amino acids, such as Lys, Leu, Met, and Asn, with low frequencies compared with other groups. Furthermore, the group D proteomes contained the largest percentages of charged and aliphatic amino acids among the 11 groups. The group E species were all Bacteria, and mainly from the Alphaproteobacteria class. The proteomes of the species in this group had the lowest frequency of Cys among the 11 groups. The group F was a large branch of Bacteria species mainly from the Actinobacteria and Proteobacteria. Arg was the dominant amino acid proteomes of the species in group F, and Glu and Ser were least abundant. Moreover, the group F proteomes had the largest portion of hydrophobic amino acids, but the smallest portion of polar and uncharged amino acids among the 11 groups. The groups G, H and I were three small branches of Bacteria species, mainly from Gammaproteobacteria (group G), Cyanobacteria (group H), and Firmicutes and Proteobacteria (group I). The dominant amino acid in the group G proteomes was Met, while, in the group H, Leu, Gln, and Trp were the dominant amino acids. The group H proteomes also had the lowest composition of charged amino acids among the 11 groups. Amino acid frequencies in the proteomes of the species in group I showed a balanced distribution, implying no obvious frequency preference. The group J and K branches contained species from both Archaea and Bacteria. In group J, Euryarchaeota and Firmicutes were the dominant species, and in group K Bacteroidetes/Chlorobi, Euryarchaeota, and Firmicutes were dominant. The group J proteomes had the lowest frequency of Thr among the 11 groups. The amino acid distribution in the group K proteomes was distinctive; Asn, Ile, Lys, Phe, and Tyr were dominant, while Ala, Arg, Pro and Trp, had the lowest frequencies among the 11 groups. Furthermore, the group K proteomes contained the highest percentages of aromatic amino acids and the lowest percentages of heterocyclic amino acids.

When we examined the correlations between the 20 amino acid frequencies and their corresponding synonymous codon frequencies in the 11 groups (Additional file 3), we found that the correlation was highest in group B, which contained only Eukaryota. In groups E, F, G, H, and I, all of which contained only Bacteria, the correlations were also high, while in group D, which contained only Archaea, the correlation was relatively low. In the other groups, which contained species from different domains, the correlation coefficients showed a divergent distribution. It is worth mentioning that, in group K, the overall amino acid frequencies appeared to be the least correlated with the synonymous codon frequencies among the 11 groups.

## Discussion

### Correlation between amino acid frequencies and their synonymous codon frequencies

Although the origin of the standard genetic code with its 20 amino acids has long been debated, it may be that the genetic code and amino acid frequencies have evolved to approach an optimal codon/amino acid relation; in other words, the genetic code may be selected to match the average optimal concentration of the amino acids [35,37-39]. Indeed, there is a clear tendency for amino acids that are encoded by more codons to be more frequent in proteomes, although, as we have shown, the strength of this relationship, as evidenced by the Pearson coefficients between amino acid frequencies and their synonymous codon frequencies, varied considerably between species (Figure 2).

For individual amino acids, the concordance was not perfect. We found that most of the individual amino acids showed an obvious bias against the rates expected from a random distribution of the 61 sense codons (Figure 1). The up group of Ala, Asn, Asp, Glu, Lys, Met, and Phe showed significantly higher frequencies than their expected rates and most of the amino acids in this group have relatively small numbers of encoding codons. The down group of Arg, Cys, His, Pro, Ser, Thr, and Trp had significantly lower frequencies than their expected frequencies and these amino acids have relatively high numbers of encoding codons. Some of the deviation from expected frequency rates can be explained as a consequence of selection pressure at the level of protein structure and function. For example, Met is principally an initiation amino acid and, therefore, its presence does not follow a random distribution, explaining why it occurs at higher than expected rates. Both Cys and Pro, strongly influence protein tertiary structure, Cys by forming disulphide bridges, and Pro by terminating helices and introducing turns [11]. If they occurred in higher proportions, protein misfolding may result. In addition, some of this variation from expected frequency rates can be explained as the result of selection pressure at the level of the nucleic acid sequences. For example, Arg is coded by six triplet codons, four of which begin with the dinucleotide CG, which is known to be suppressed in many classes of organisms such as vertebrates, and in many diverse species from protist, dicot plants and bacteria [11,29]. This characteristic may lead to the suppression of codons for Arg and, hence, to its underrepresentation. In their study, Bharanidharan et al. [11] reported that the variations of Arg, Val, Asp, Glu, Ser, and Cys from predicted values were attributable to selection pressure at the amino acid level, while the variations of Thr, Phe, Lys, and Asn arose only from mutation and selection pressure at the level of the nucleic acid sequences.

For some amino acids, however, the variations from the expected frequency cannot be easily explained by these reasons. In our view, the deviation of some amino acids may not have arisen from themselves, but from strong preferences for other amino acids. In other words, when some amino acids are favored or rejected, this bias is bound to affect the frequencies of the other amino acids, even with no bias from them.

### Effect of GC pressure on the amino acid compositions and on the framework of amino acid properties in the proteomes

**Figure 6:**
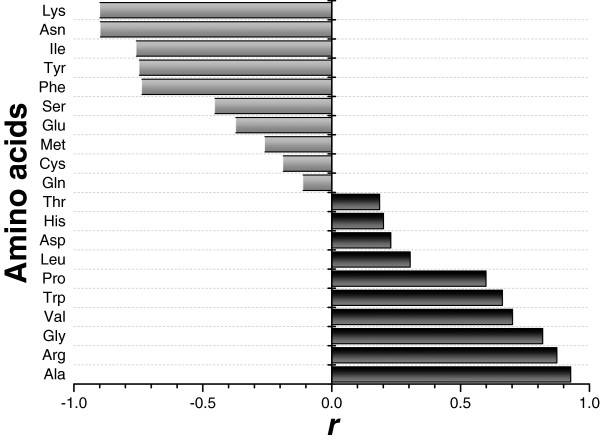
**Correlation between genomic GC content and amino acid frequency across the 461 species.** The Pearson correlation coefficient (*r*) was calculated between amino acid frequencies and genomic GC content. The *p* values distribution was *p* = 0.0265 (Gln) and *p* < 0.0001 (others).

The correlations of the frequencies of some of the amino acids were statistically significant (Additional file 1). Rather intriguingly, the amino acids in the two distinct groups (group one with Ala, Arg, Gly, Pro, Trp and Val; and group two with Asn, Lys, Ile, Phe and Tyr) were positively correlated within a group, but negatively correlated between the groups. When we looked for the biological driving force that might be behind this statistical association, we observed that all the amino acids in the group one except Val are coded by GC-rich codons, whereas the amino acids in the group two are coded by AT-rich codons. Previous studies have indicated that variations in the nucleotide composition, as measured by the GC content, have a significant impact on amino acid composition. Thus, the use of amino acids encoded by GC-rich codons increased with increasing genomic GC content, and the use of amino acids coded by AT-rich codons decreased with increasing GC content. The frequencies of amino acids encoded by neutral codons were not affected by changes of genomic GC content [6,9-14]. Therefore, we measured the correlation between the proportion of amino acids in the proteomes and the genomic GC content across the species in the three domains (Figure 6). We observed that the frequencies of the amino acids in the group one showed significant positive correlations with the genomic GC content, while the frequencies of the amino acids in the group two displayed strong negative correlations with the genomic GC content. Therefore, we inferred that GC pressure was the main force that maintained the association of these amino acids.

Surprisingly, not only was amino acid composition affected by nucleotide bias, but the effect was large and widespread among the three domains. The genomic GC content across all the tested species ranged from 25 to 75%. For each 10% increase in genomic GC content, the use of Ala, Arg, Gly, Pro, Trp, and Val increased by 2.05, 1.04, 0.85, 0.52, 0.14, and 0.59%, respectively, while the use of Asn, Lys, Ile, Phe, and Tyr decreased by 1.02, 1.62, 1.16, 0.41 and 0.47%, respectively.

Next, we performed a correlation analysis between the distribution of amino acid properties and the genomic GC content across all the species to find whether GC variation would affect the framework of the amino acid properties (Additional file 4). We found that genomic GC content showed a strong negative correlation with the aromatic amino acids and a strong positive correlation with the hydrophobic amino acids. These correlations can be explained by the amino acids in both these groups. In the aromatic group, the main contributors, Phe and Tyr, were negatively correlated with the GC content, while in the hydrophobic group, the main contributors, Ala, Pro, Trp, and Val, were positively correlated with the GC content (Figure 6). However, we also observed that the impact of genomic GC content on the global physico-chemical trends of the amino acids seemed to be limited. For each 10% increase in genomic GC content, the proportion of aromatic amino acids decreased by 0.74%, while the proportion of hydrophobic amino acids increased by 1.88%. Thus, the change of the physico-chemical groups with genomic GC content was relatively low compared with that of the individual amino acids. In addition, the genomic GC variation showed no significant correlations with other important physico-chemical groups (Additional file 4). Therefore, we hypothesized that the framework of the amino acid properties in the proteomes was in a relatively stable state, and, at least, able to withstand GC pressure. Accordingly, two challenging questions arose. One, is the relatively stable physico-chemical framework in the proteomes the material basis of species in response to the pressure for survival? And two, can the use of amino acids be substituted by amino acids with similar physico-chemical properties? Further research on the roles that the physico-chemical properties play in the protein structure and function is needed to address the first question. As for the second question, despite our proposal that genomic GC variation largely affects the use of many of the amino acids, the physico-chemical framework was relatively independent of genomic GC content because of the frequency complementation between the GC-dependent amino acid pairs in the physico-chemical groups. For example, the frequency of amino acid Arg was positively correlated with genomic GC content while the frequency of Lys was negatively correlated (Figure 6); therefore, the GC-dependent amino acids Arg and Lys are complementary pairs in charged group and probably, the use of Arg and Lys was interchangeable to a certain degree. This finding might provide some evolutionary clues on amino acid substitutions. Indeed, it is worth exploring the biological significance of a stable physico-chemical framework of amino acids by the complementation of GC-dependent amino acids.

### Principal components of amino acid matrices may represent GC variation, domain discrimination, and environmental influences

To extract information about the associations between the amino acid features and the corresponding observations from the data matrices, we used principal component analysis based on the projection of the 20 dimensional frequency vectors of the 20 standard amino acids onto their salient aspects. The most significant principal component (Prin1) in the analysis accounted for 43.91% of the total information in the matrices. In Prin1, the GC-dependent amino acids, Ala, Arg, Gly, Pro, Trp, and Val, were concentrated at high values, whereas Ala, Arg, Gly, Pro, Trp, and Val displayed low values (Figure 6 and Additional file 2). Prin1 showed a highly significant correlation to the genomic GC content (*R* = 0.9394, *p* < 0.0001), indicating that, to a large extent, genomic GC content determined amino acid usage. This factorial axis was assigned as the “GC-axis”. This finding was similar to earlier observations for numerous genomes that GC content was the most important determinant of global amino acid composition despite differences in information volume [3,16,17,24]. The observed consistency of the impact of genomic GC content on patterns of amino acid composition across all three domains supports the idea that genomic GC content may be a driving force in genome evolution [12].

In contrast, Prin2, which represented 16.84% of the total information, separated the Archaea, Bacteria, and Eukaryota domains (Figure 4). Accordingly, this factorial axes was responsible mainly for the domain discrimination. The main amino acids were Gln, His, Ser, and Val. In previous studies, the second factorial axis was assimilated to a temperature axis because it could distinguish temperature-sensitive species [3,4,24]. Interestingly, in previous studies, these four amino acids were identified as amino acid signatures of hyperthermophiles, indicating that Prin2 shared a similar structure despite minor differences in the amino acid signatures arising from differences in the scope of the tested species [3,4,17,24]. However, it should be stressed that Prin2 was responsible for domain discrimination rather than growth temperature, because the separation was more related to taxonomic class than to the temperature preferences of the species. For example, the frontiers between Eukaryota and the other domains were clear in this factorial axis (Figure 4). Consequently, we proposed that Prin2 was subjected to the underlying amino acid preferences in the different taxonomic domains. We showed that the distribution of Gln, His, Ser, and Val had distinguishing features between the three domains (Figure 1). For example, the occurrence of Gln and Val was significantly lower in Archaea compared with the other two domains, while the occurrence of Ser was remarkably higher in Eukaryota compared with the other two domains. The occurrence of His also showed a distinguishable distribution in the three domains. Therefore, we suggested that the obvious preferences of these amino acids in Prin2 were in response to the domain discrimination.

Prin3 accounted for 11.06% of the variance in the multivariate analysis and its amino acid signature contained Cys, Leu, and Thr (Additional file 2). Prin3 appeared to be related with environmental niches. In Archaea, the Prin3 values decreased from hyperthermophiles (Prin3 values of 3.3–1.6; representative species *Desulfurococcus fermentans*, *Pyrobaculum aerophilum*, *Pyrococcus abyssi*, *Thermococcus barophilus*, and *Thermoproteus tenax*) to acidophiles (Prin3 values of 1.6–1.4; representative species *Thermoplasma acidophilum* and *Sulfolobus acidocaldarius*) to mesophiles or anaerobes (Prin3 values of 1.1–-0.2; representative species *Methanococcus aeolicus* and *Methanocorpusculum labreanum*) to psychrophiles (Prin3 values of around −0.3; representative species *Cenarchaeum symbiosum*) to halophiles (Prin3 values of − 0.3– − 3.5; Halobacteria species). Accordingly, in Bacteria, the thermophiles (representative species *Anaerolinea thermophila*, *Meiothermus ruber*, *Thermodesulfatator indicus* and *Thermus aquaticus*) also gathered at highest end, but with higher Prin3 values (5.9–3.2) than in Archaea. For the Eukaryota species, especially the higher animals and plants, it was more difficult to determine the environmental conditions that could be correlated directly with Prin3 because of the many differences in their morphology and living environments. Interestingly, previous studies have suggested that the amino acid composition of the proteomes in endothermic vertebrates was biased in the same direction as the amino acid composition in thermophilic bacteria and archaea [26]. Together, these results might suggest that Prin3 is subjected to a complex mixture of environmental influences such as growth temperature, pH, oxygen, and solvent, which may provide important new information to refine the global picture of lifestyles and genomes.

### Association of amino acid composition dendrogram with genomic GC content, phylogeny, and lifestyles

To compare amino acid composition across the species, organisms with similar compositions for all 20 amino acids were clustered using a neighbor-joining method with distances computed using the Euclidean metric on a dataset that consisted of the overall percent amino acid composition for each of the 461 organisms. The resulting dendrogram had 11 main branches (Figure 5). Comparisons between branches indicated that amino acid composition was associated closely with genomic GC content, phylogeny, and environmental factors. Generally, organisms within a cluster had a similar genomic GC content. However, it should be noted that organisms with similar GC content were not necessarily proximate neighbours. It was worth mentioning that the group F proteomes had the highest GC content (average of 63%) and group E proteomes had the lowest GC content (average of 36%).

Most Eukaryota were in group B, which reflected a distinctive feature of the amino acid architecture of their proteomes, while Archaea and Bacteria revealed general differences in their amino acid composition. The differentiation of the three domains was reflected more obviously in Prin2 (Figure 4). Previous studies have also revealed that proteomic composition could generally discriminate among archaea, bacteria, and eukaryotes [4,6,16]. However, interlinkages between proteomic amino acid composition and species phylogeny were limited in characterizing the species diversity. The frequency distributions of the 20 amino acids among the different taxa were compared (Additional file 5). In general, on the one hand, it was common for remote species, such as those in the groups Crenarchaeota and Synergistetes, to share a similar amino acid distribution. On the other hand, species in the phylogenetically related group might also show considerable differences. For example, species in the group Spirochaetes displayed large differences in the amino acids frequencies, as reflected by the CV values. Therefore, information about the amino acid composition of the proteomes was insufficient to reflect the detailed species classification, probably because of the limited information capacity of 20-dimensional amino acid data.

Organisms that share similar lifestyles tended to be grouped together by amino acid composition clustering. For instance, halophiles were clustered in group D while groups C and J consisted mainly of archaea and bacteria hyperthermophiles. Interestingly, a clear distinction was observed between the species in these two groups: species with a genomic GC content around 53% were clustered in group C, and species with a relatively low genomic GC content (around 44%) were clustered in group J. This finding is not surprising because genomic GC content was found to show no correlated response to optimal growth temperatures [40,41].

### Effect of genomic GC content, phylogeny, and lifestyles on frequency concordance of the genetic code and amino acids

**Figure 7:**
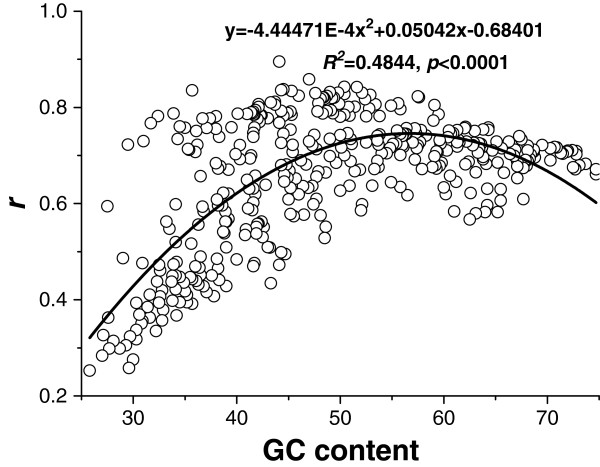
**Relationship between genomic GC content and concordance of overall amino acid composition and sense codons.** The concordance was weighed by the Pearson correlation coefficient (*r*) between the frequencies of the 20 amino acids and the corresponding synonymous codon frequencies based on the universal genetic code. A parabolic model was used to simulate the trend of the concordance against GC content.

Amino acid composition was affected greatly by genomic GC content. Therefore, we examined the relationship between genomic GC content and frequency concordance of the genetic code and amino acids (Figure 7). The general trend showed that as the genomic GC content increased from 25 to 75%, the concordance first enhanced and then declined. In other words, the concordance weakened at the extremes of the genomic GC content (too high or too low). The relationship was fitted to a parabolic model and the significant fitting (*R*^*2*^ = 0.4844, *p* < 0.0001) indicated that the concordance reached a peak at a genomic GC content of around 56.7%.

**Figure 8:**
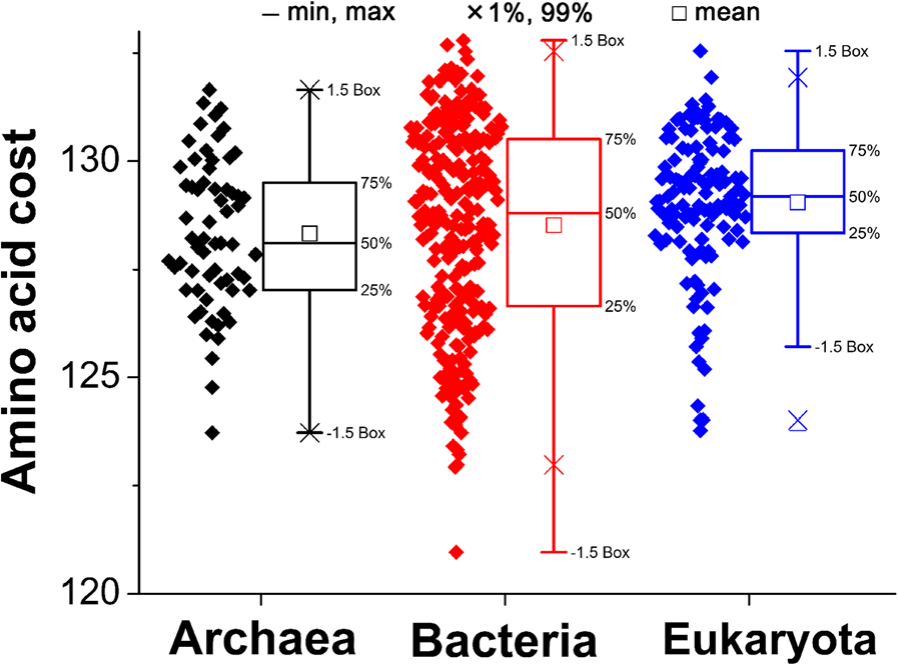
**Comparison of the distribution of amino acid biosynthetic costs among the three domains.** The amino acid biosynthetic cost was as reported by Seligmann [47]. Briefly, the molecular weight was used as a proxy for biosynthetic cost, which has the advantage in comparisons across species of being independent of the pathways for amino acid synthesis [46,47]. The cost in each species was taken as the accumulation of the costs of the 20 amino acid costs (amino acid percentage multiplied its molecular weight).

The concordance was also associated with species phylogeny. Interestingly, more concordance between the genetic code and amino acid composition was found in Eukaryota than in the other two domains (Figure 2). In this study, the concordance differences between prokaryotes and eukaryotes could be explained as the balance between metabolic efficiency and energy cost in protein biosynthesis. The analyzed prokaryotes are unicellular while eukaryotes are mostly multicellular. Interestingly, like the prokaryotes, the unicellular eukaryotes, for example the yeasts, also had a low correlation between amino acid frequency and synonymous codon frequency (lowest end of Figure 2). Multicellular eukaryotes, however, had a higher correlation of amino acid frequency and synonymous codon frequency. It is possible that unicellular organisms have a relatively low recycling efficiency of amino acids because of their more direct interaction with the external environment; for example, extracellular proteins and surface proteins of microbes are less likely to be recycled by the cell or passed down during cell division [42]. Moreover, unicellular organisms may have a relatively lower ability to complete for limited resources. Previous studies have indicated that unicellular microbes tended to undergo economical evolution at the level of an entire protein or even an entire proteome by, for example, preferentially using less biosynthetically expensive amino acids in their highly expressed proteins and extracellular proteins to counteract the potential loss of cellular resources [34,42-45]. Our analyses have shown that, compared with Eukaryota, the Archaea and Bacteria proteomes contain more of the inexpensive amino acids like Ala, Gly, Ile, and Val, and fewer of the expensive residues like Gln, His, and Trp (Figure 1) [46,47]. These amino acid frequencies in Archaea and Bacteria were further from the expected rates than those in Eukaryota. Moreover, Archaea and Bacteria showed the relative economy of proteomes (Figure 8). Generally, the costs of the proteomes in descending order were Eukaryota, Bacteria, and Archaea, which is consistent with the descending order of the concordance: Eukarya, Bacteria and Archaea (Figures 2 and 8). On the whole, Eukaryota may care more about the metabolic efficiency since the high concordance may improve the metabolic efficiency in protein biosynthesis. Comparatively, Archaea and Bacteria may likely favor the use of cheaper, but less efficient amino acids, which lead to the relatively low concordance that we observed. From the evolutionary perspective, multicellular Eukaryota may have moved toward a high concordance for metabolic efficiency because the recycling efficiency of their amino acids is high and they have a relatively strong viability for more energy, while the unicellular prokaryotes may have moved toward the more economical evolution of proteomes to save energy and cellular resources.

Species with similar lifestyles tended to cluster and to have similar concordance. For example, the hyperthermophiles in group C reflected a high adaptability between overall amino acid frequencies and the encoding codon frequencies with correlation coefficients (*r*) around 0.7, while halophiles in group D showed medium adaptability with correlation coefficients (*r*) around 0.6 (Additional file 3). This finding suggested that the lifestyles of species may also affect the concordance of the genetic code and amino acids.

## Conclusions

In this study, the overall composition, chemical architecture, frequency relationship, main component, and distribution features of the 20 standard amino acids in the proteomes of 461 species from the three domains of life were analyzed systematically. Our results and previous observations together point to the idea that proteomic architecture has been shaped by the integrated forces of GC pressure, phylogeny, and environmental niche during evolution. These findings could contribute to our understanding of proteome evolution, and even to biological evolution.

GC content is the most important determinant of global amino acid composition. In particular, GC content has significant positive effects on the frequencies of Ala, Arg, Gly, Pro, Trp, and Val, and negative effects on the frequencies of Asn, Lys, Ile, Phe, and Tyr. Except for Val, the other GC-dependent amino acids are encoded either by GC-rich or AT-rich codons. However, physico-chemical framework of proteomes is relatively independent against GC pressure through a frequency complementation of GC-dependent amino acid pairs with similar physico-chemical property. On the one hand, our results suggested that a relatively stable physico-chemical framework might be the material basis of the response of species to GC pressure during evolution. On the other hand, the results implied that the use of complementary pairs such as Arg and Lys might be in mutual substitution, which, to a certain degree, is attributable to their similar physico-chemical properties, to buffer the influence of GC pressure on protein structure and function.

Species phylogeny is the second most important factor that influenced proteomic architecture. The cardinal amino acids are Gln, His, Ser and Val, and the constituted component could discriminate Archaea, Bacteria, and Eukaryotes from each other. This finding suggested that the different species phylogenies could be traced based on the proteomic architecture during evolution as early as the divergence of the three domains. Consequently, the proteomic architecture vector might serve as an additional useful tool for understanding taxonomic differences.

Environmental niche brewing a series of habitat factors, such as growth temperature, pH, oxygen and solvent, is also a significant factor to determine the proteomic architecture especially for archaea and its amino acid signature is Cys, Leu and Thr. The environmental component could clearly differentiate lifestyle groups such as hyperthermophiles, acidophiles, mesophiles, and halophiles among the unicellular prokaryotes. However, the relatedness between environmental influences and the proteomes of multicellular eukaryotes was rather weak. It is likely that multicellular higher organisms have a greater capacity to respond to physiological constraints. In other words, external factors have limited impacts on the intracellular environment in multicellular eukaryotes compared with their impact in unicellular prokaryotes, and this ability to respond probably arose from evolutionary resistance to environmental pressure. These results may provide important new information to refine the global picture of the relationships between lifestyles and genomes.

We also found that the frequency concordance of the genetic code and amino acids was not perfect for an individual amino acid. Most of the individual amino acids showed a statistically significant bias against the rates expected from a random distribution of the 61 sense codons. In general, Ala, Asn, Asp, Glu, Lys, Met, and Phe showed significantly higher frequencies than their expected rates, while Arg, Cys, His, Pro, Ser, Thr, and Trp showed significantly lower frequencies. The deviation of some amino acids such as Arg, Cys, Met, and Pro was explained as a consequence of selection pressure at the level of the nucleic acid sequences or protein structure and function. However, we also proposed that the deviation of some amino acids may not have arisen from themselves, but from other amino acids. When certain amino acids are favored or rejected, the frequencies of other amino acids will be affected, even with no bias from them. Overall, the concordance of the amino acids was associated closely with genomic GC content, phylogeny, and lifestyles. Extreme genomic GC contents weakened the concordance and the results suggested that concordance preferentially occurred at a genomic GC content around 56.7%. The concordance in Eukaryota was significantly better than in the other two domains, which we explained as a balance between metabolic efficiency and energy cost in protein biosynthesis. The concordance also differed between species with similar habitats; for example, hyperthermophiles reflected a higher adaptability than halophiles. The observations reported here may promote the understanding of co-adaptation between proteomic architecture and the genetic code.

## Methods

### Sequence data

A total of 461 species covering the Archaea, Bacteria, and Eukaryota domains were used in this study (Additional file 6). The protein sequences from the proteomes of each of the species were extracted from the NCBI Genome database (http://www.ncbi.nlm.nih.gov/genome/browse/).

### Analysis of amino acid composition

The percentages of the 20 standard amino acids in the proteomes of each species were calculated by parsing the overall protein sequences. The 20 amino acids were classified into hydrophobic (Ala, Ile, Leu, Met, Phe, Pro, Trp, and Val), charged (Arg, Asp, Glu, His, and Lys), and polar and uncharged amino acids (Asn, Cys, Gln, Gly, Ser, Thr, and Tyr) according to the polarity of their side chains. The 20 amino acids were also divided into aliphatic (Ala, Arg, Asn, Asp, Cys, Gln, Glu, Gly, Ile, Leu, Lys, Met, Ser, Thr, and Val), aromatic (Phe, Trp, and Tyr), and heterocyclic (His and Pro) amino acids based on the chemical structure of their side chains [10,48]. The proteomes were analyzed by summing the respective contributions of the corresponding amino acids under each of the classifications.

### Construction of a clustering tree

The cluster analysis of the proteomes from the 461 species was based on their different amino acid compositions. The Euclidean distance of the amino acid composition was used to reflect the distance between any two species. A neighbor-joining tree was constructed from the distance data using Mega 5.05 [49]. To view the phylogenetic trees and draw the figures, the tree cluster data were submitted to iTOL (http://itol.embl.de/upload.cgi) [50].

### Statistical analysis

All the data analyses in this study were performed using SAS statistic software (SAS Institute Inc., Cary, North Carolina, USA). The normality of amino acid frequencies was performed with the Shapiro-Wilk test; Pearson correlation coefficients were used to evaluate the correlation between amino acid frequencies; comparative analysis among the amino acid frequencies was performed at the 0.05 level using Duncan's multiple-range test; and principal component analysis was performed to explore the data matrix of amino acid frequencies.

## Competing interests

The authors declare that they have no competing interests.

## Authors’ contributions

WC, YS, and FC conceived and designed the research. WC conducted the bioinformatics analyses. WC and FC wrote the paper. All authors read and approved the final manuscript.

## Supplementary Material

Additional file 1 **Correlation coefficients between the frequencies of 11 amino acids in the three domains.** The correlation between 20 standard amino acid frequencies was studied. Pearson correlation coefficients were used to evaluate the correlation between amino acid frequencies. The figure presents the correlations of 11 amino acids, which had significant correlations in frequency. Click here for file

Additional file 2 **Principal component analysis of the 20-dimentional amino acid frequency matrix.** Prin1, Prin2 and Prin3 were the top three components, accounting for 43.91%, 16.84% and 11.06% of the total information, respectively. (a) Factorial plane of Prin1 and Prin2. (b) Factorial plane of Prin1 and Prin3. (c) Factorial plane of Prin2 and Prin3. Click here for file

Additional file 3 **Comparison of the features of amino acid distribution in the 11 main branches of the clustering tree.** The values were expressed as averages and reflected by rectangle lengths. (a) The frequency distribution of the 20 amino acids in the 11 main branches (groups A–K). (b) The frequency distribution of charged, hydrophobic, and polar and uncharged amino acids. (c) The frequency distribution of aliphatic, aromatic and heterocyclic amino acids. (d) Boxplot of Pearson correlation coefficients between the 20 amino acid frequencies and their corresponding synonymous codon frequencies in the 11 groups. Click here for file

Additional file 4 **Correlation analysis of physico-chemical properties of amino acids with genomic GC content in 461 species.** Pearson correlation coefficients were used to evaluate the correlation between composition of physico-chemical groups and genomic GC content in 461 species. Click here for file

Additional file 5 **Comparison of the distribution of the 20 amino acid frequencies in different taxonomic groups.** Archaea groups (Crenarchaeota, Euryarchaeota, Korarchaeota, Nanoarchaeota and Thaumarchaeota), Bacteria groups (Actinobacteria, Aquificae, Bacteroidetes/Chlorobi, Chlamydiae/Verrucomicrobia, Chloroflexi, Chrysiogenetes, Cyanobacteria, Deferribacteres, Deinococcus-Thermus, Dictyoglomi, Elusimicrobia, Fibrobacteres/Acidobacteria, Firmicutes, Fusobacteria, Gemmatimonadetes, Nitrospirae, Planctomycetes, Proteobacteria, Spirochaetes, Synergistetes, Tenericutes, Thermode sulfobacteria and Thermotogae) and Eukaryota groups (Animals, Fungi, Plants and Protists). The taxonomy information was extracted from NCBI classification. The amino acid frequencies in different taxonomic groups were represented as averages plus standard deviations and their expected values based on the universal genetic code were indicated by dash lines. Click here for file

Additional file 6 List of the 461 species from Archaea, Bacteria, and Eukaryota analyzed in this study. Click here for file
