# Supplementary material for: Evolution of complete proteomes: guanine-cytosine pressure, phylogeny and environmental influences blend the proteomic architecture
Source: BMC Evol Biol. 2013 Oct 3;13:219. doi: 10.1186/1471-2148-13-219 (PMC3850711; doi:10.1186/1471-2148-13-219)
Supplement: Additional file 1 — Correlation coefficients between the frequencies of 11 amino acids in the three domains. The correlation between 20 standard amino acid frequencies was studied. Pearson correlation coefficients were used to evaluate the correlation between amino acid frequencies. The figure presents the correlations of 11 amino acids, which had significant correlations in frequency. [file 1471-2148-13-219-S1.pdf]

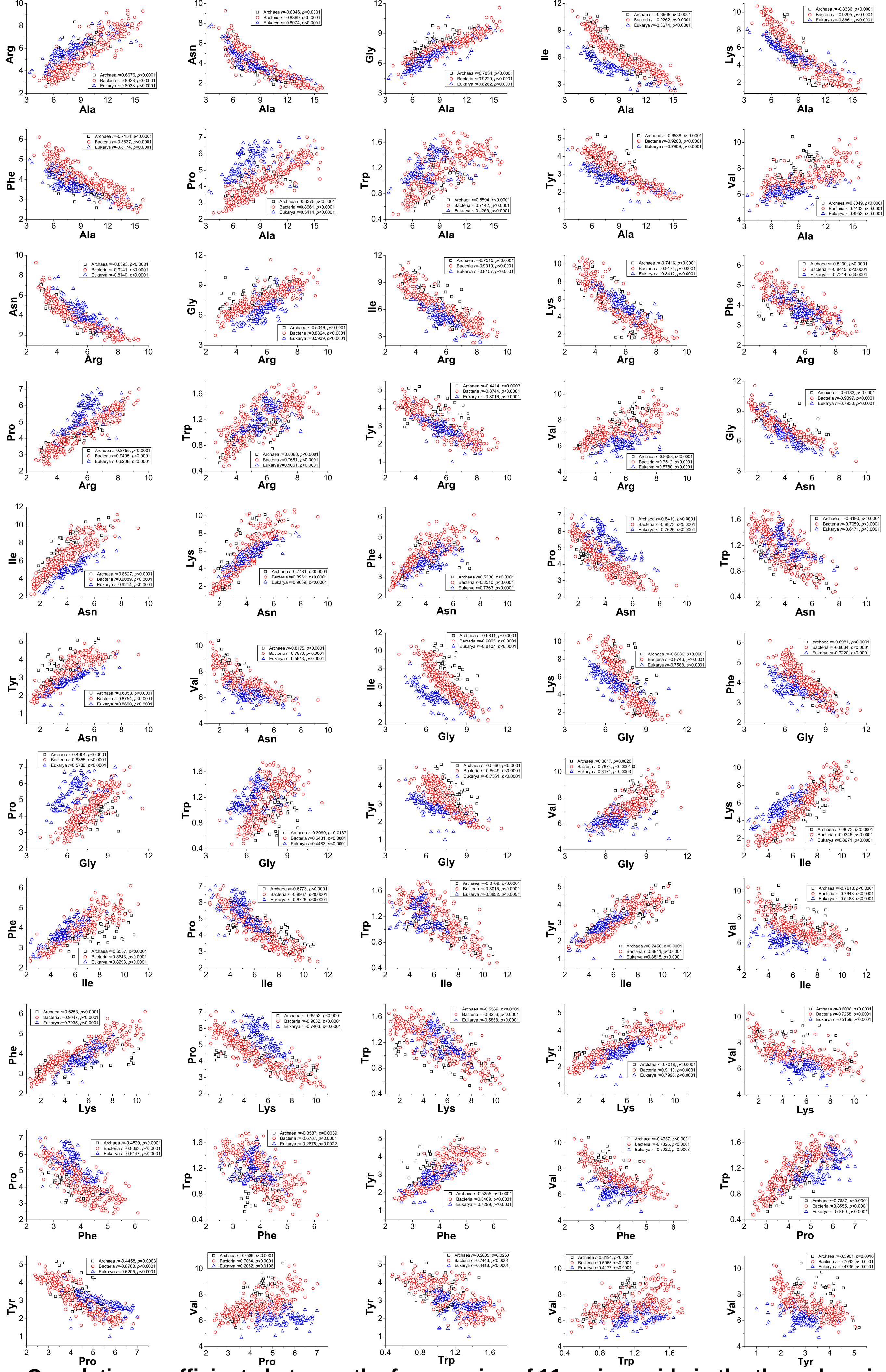

Correlation coefficients between the frequencies of 11 amino acids in the three domains

The correlation between 20 standard amino acid frequencies was studied. Pearson correlation coefficients were used to evaluate the correlation between amino acid frequencies. The figure presents the correlations of 11 amino acids, which had significant correlations in frequency.
