# Supplementary material for: Evolution of complete proteomes: guanine-cytosine pressure, phylogeny and environmental influences blend the proteomic architecture
Source: BMC Evol Biol. 2013 Oct 3;13:219. doi: 10.1186/1471-2148-13-219 (PMC3850711; doi:10.1186/1471-2148-13-219)
Supplement: Additional file 2 — Principal component analysis of the 20-dimentional amino acid frequency matrix. Prin1, Prin2 and Prin3 were the top three components, accounting for 43.91%, 16.84% and 11.06% of the total information, respectively. (a) Factorial plane of Prin1 and Prin2. (b) Factorial plane of Prin1 and Prin3. (c) Factorial plane of Prin2 and Prin3. [file 1471-2148-13-219-S2.pdf]

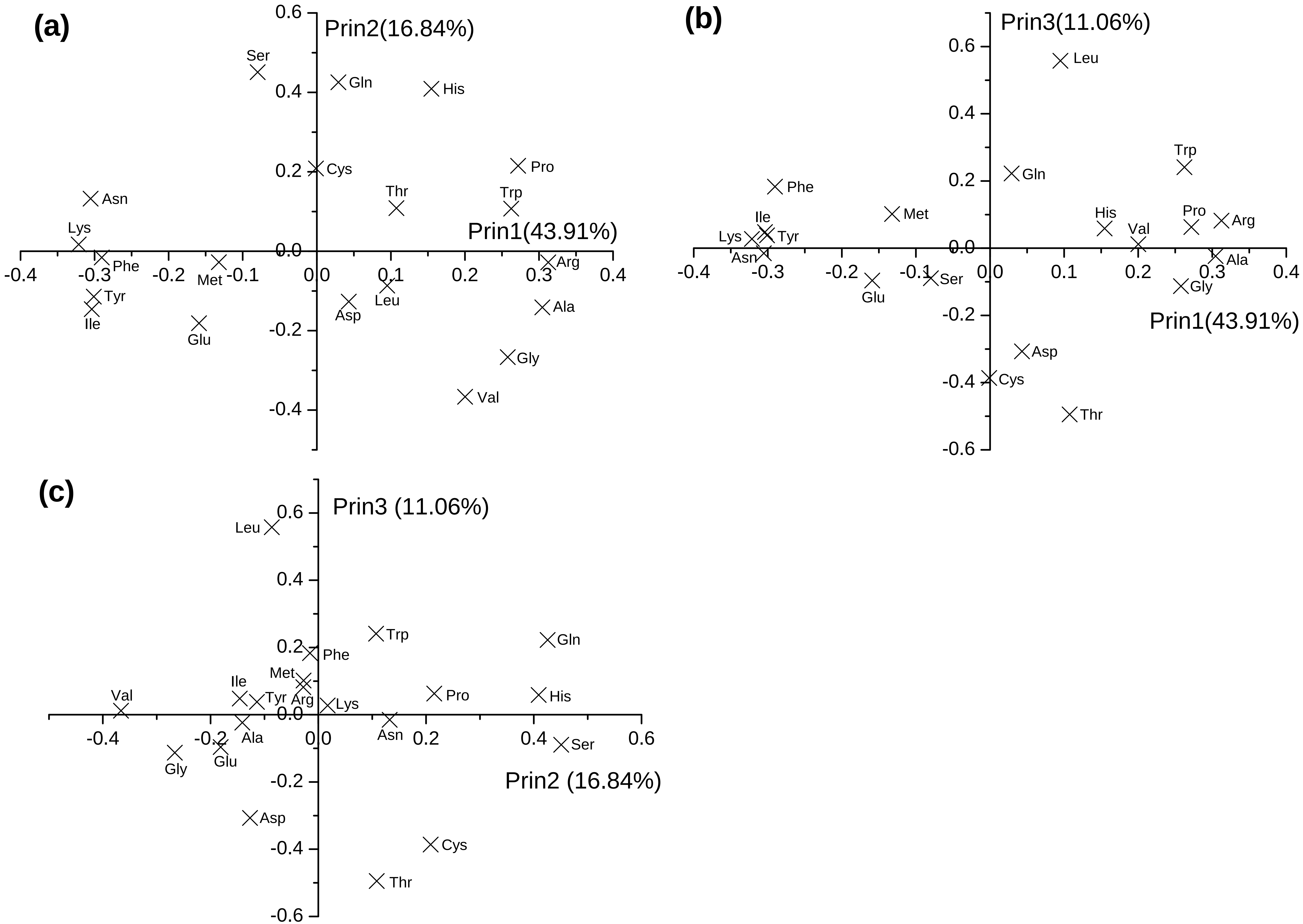

Principal component analysis of the 20-dimentional amino acid frequency matrix. Prin1, Prin2 and Prin3 were the top three components, accounting for 43.91%, 16.84% and 11.06% of the total information, respectively. (**a**) Factorial plane of Prin1 and Prin2. (**b**) Factorial plane of Prin1 and Prin3. (**c**) Factorial plane of Prin2 and Prin3.
