# Supplementary material for: Evolution of complete proteomes: guanine-cytosine pressure, phylogeny and environmental influences blend the proteomic architecture
Source: BMC Evol Biol. 2013 Oct 3;13:219. doi: 10.1186/1471-2148-13-219 (PMC3850711; doi:10.1186/1471-2148-13-219)
Supplement: Additional file 3 — Comparison of the features of amino acid distribution in the 11 main branches of the clustering tree. The values were expressed as averages and reflected by rectangle lengths. (a) The frequency distribution of the 20 amino acids in the 11 main branches (groups A–K). (b) The frequency distribution of charged, hydrophobic, and polar and uncharged amino acids. (c) The frequency distribution of aliphatic, aromatic and heterocyclic amino acids. (d) Boxplot of Pearson correlation coefficients between the 20 amino acid frequencies and their corresponding synonymous codon frequencies in the 11 groups. [file 1471-2148-13-219-S3.pdf]

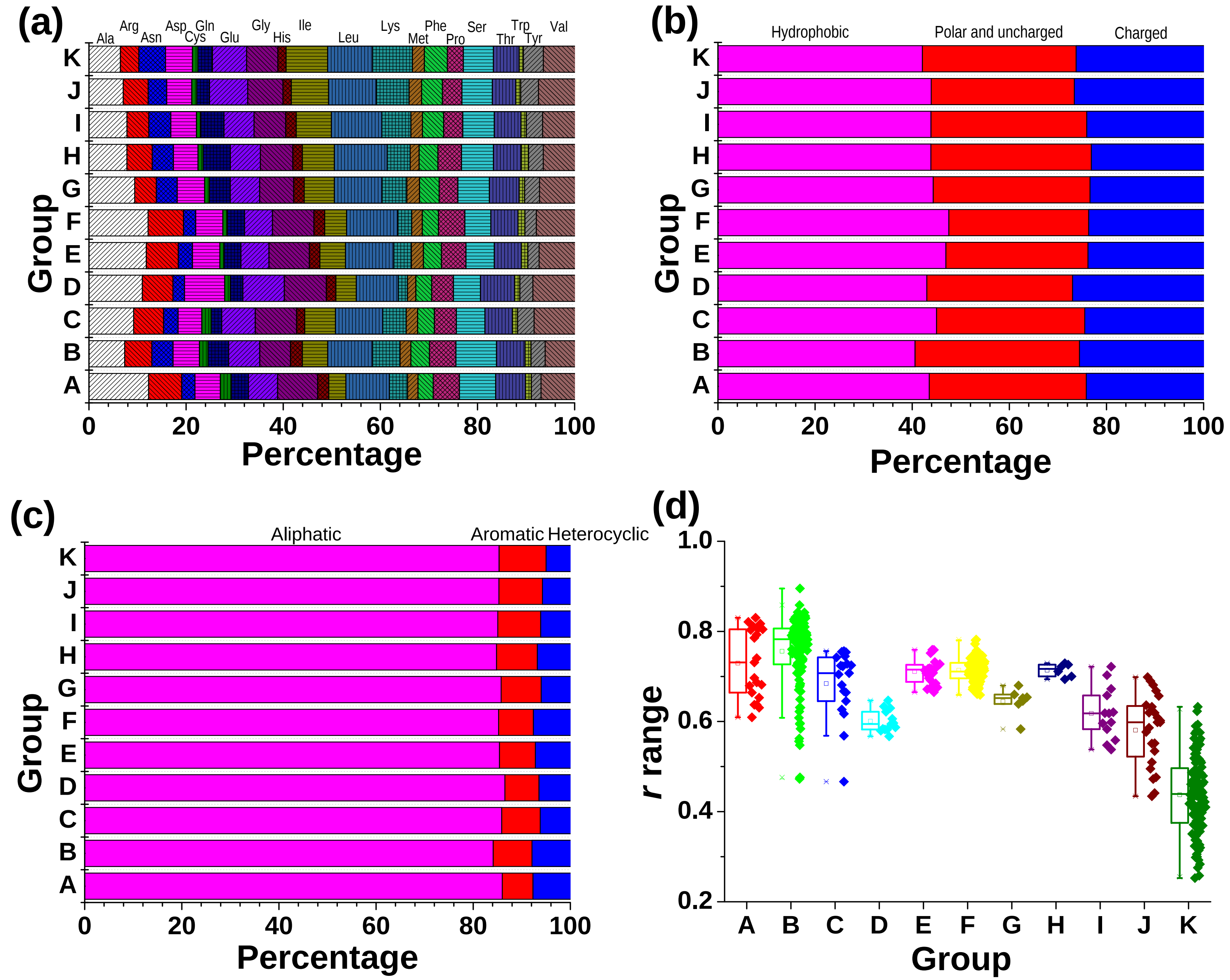

Comparison of the features of amino acid distribution in the 11 main branches of the clustering tree. The values were expressed as averages and reflected by rectangle lengths. (**a**) The frequency distribution of the 20 amino acids in the 11 main branches (groups A–K). (b) The frequency distribution of charged, hydrophobic, and polar and uncharged amino acids. (c) The frequency distribution of aliphatic, aromatic and heterocyclic amino acids. (d) Boxplot of Pearson correlation coefficients between the 20 amino acid frequencies and their corresponding synonymous codon frequencies in the 11 groups.
