# Supplementary material for: Evolution of complete proteomes: guanine-cytosine pressure, phylogeny and environmental influences blend the proteomic architecture
Source: BMC Evol Biol. 2013 Oct 3;13:219. doi: 10.1186/1471-2148-13-219 (PMC3850711; doi:10.1186/1471-2148-13-219)
Supplement: Additional file 4 — Correlation analysis of physico-chemical properties of amino acids with genomic GC content in 461 species. Pearson correlation coefficients were used to evaluate the correlation between composition of physico-chemical groups and genomic GC content in 461 species. [file 1471-2148-13-219-S4.pdf]

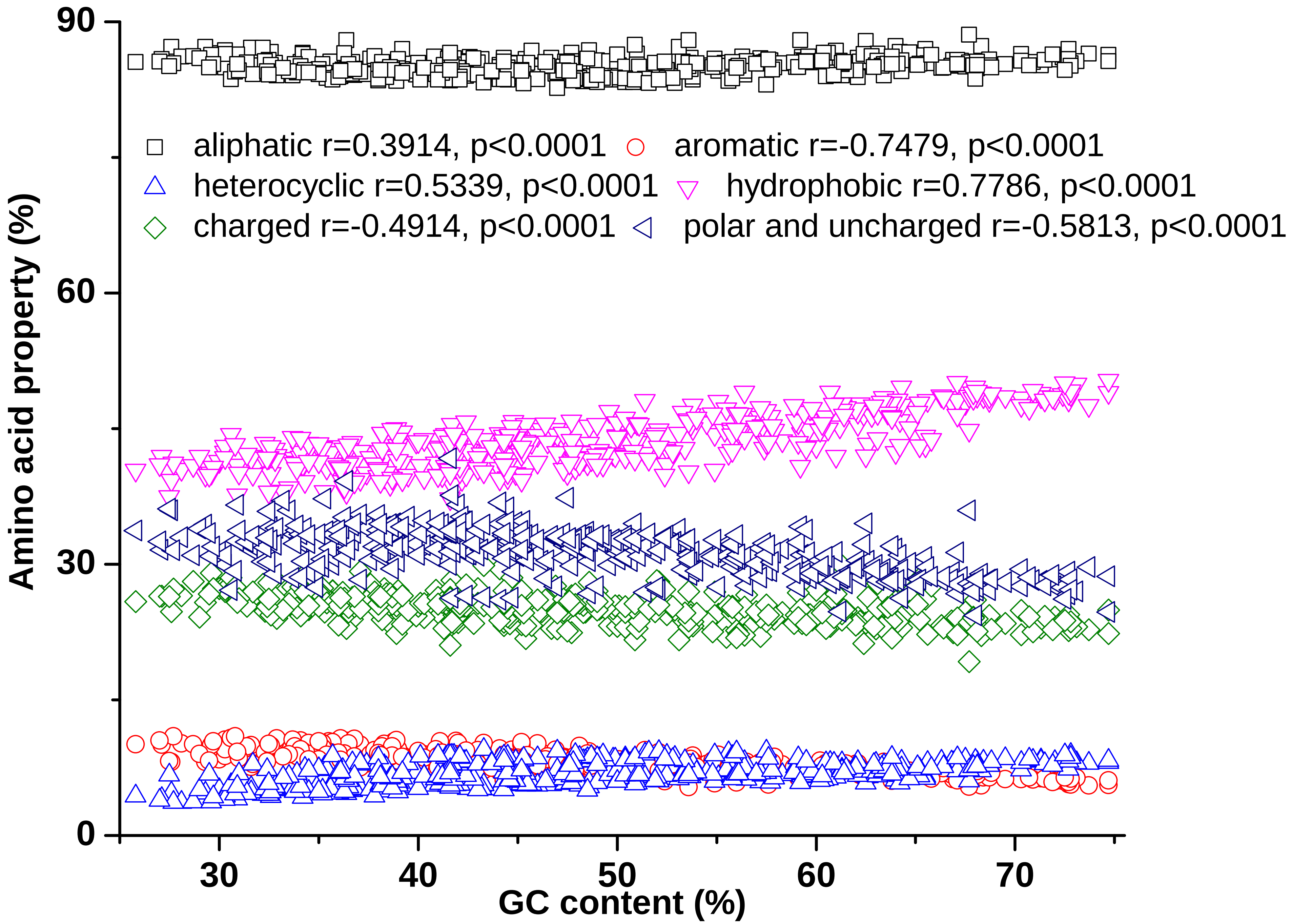

Correlation analysis of physico-chemical properties of amino acids with genomic GC content in 461 species. Pearson correlation coefficients were used to evaluate the correlation between composition of physico-chemical groups and genomic GC content in 461 species.
