# Supplementary material for: Evolution of complete proteomes: guanine-cytosine pressure, phylogeny and environmental influences blend the proteomic architecture
Source: BMC Evol Biol. 2013 Oct 3;13:219. doi: 10.1186/1471-2148-13-219 (PMC3850711; doi:10.1186/1471-2148-13-219)
Supplement: Additional file 5 — Comparison of the distribution of the 20 amino acid frequencies in different taxonomic groups. Archaea groups (Crenarchaeota, Euryarchaeota, Korarchaeota, Nanoarchaeota and Thaumarchaeota), Bacteria groups (Actinobacteria, Aquificae, Bacteroidetes/Chlorobi, Chlamydiae/Verrucomicrobia, Chloroflexi, Chrysiogenetes, Cyanobacteria, Deferribacteres, Deinococcus-Thermus, Dictyoglomi, Elusimicrobia, Fibrobacteres/Acidobacteria, Firmicutes, Fusobacteria, Gemmatimonadetes, Nitrospirae, Planctomycetes, Proteobacteria, Spirochaetes, Synergistetes, Tenericutes, Thermode sulfobacteria and Thermotogae) and Eukaryota groups (Animals, Fungi, Plants and Protists). The taxonomy information was extracted from NCBI classification. The amino acid frequencies in different taxonomic groups were represented as averages plus standard deviations and their expected values based on the universal genetic code were indicated by dash lines. [file 1471-2148-13-219-S5.pdf]

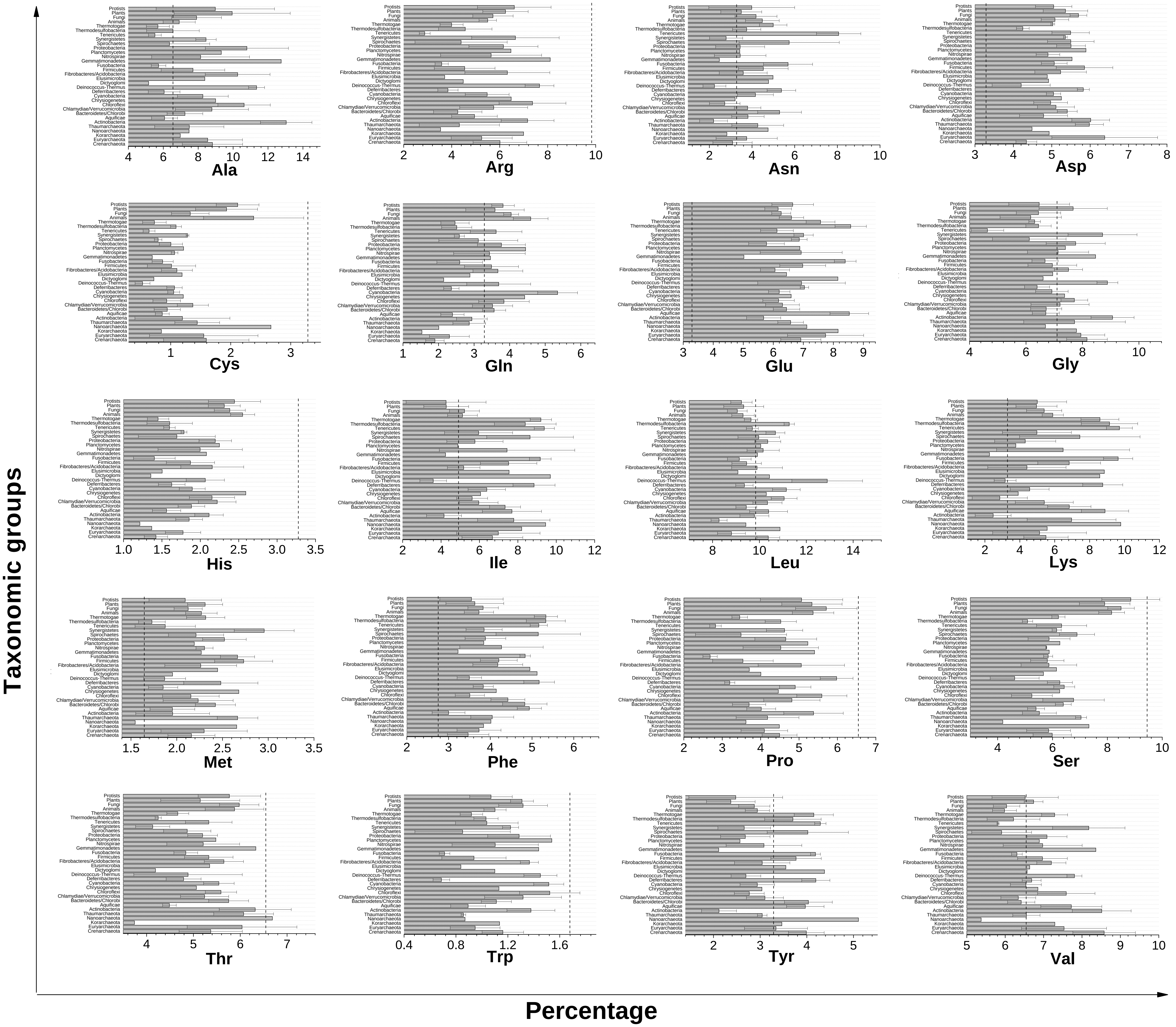

Comparison of the distribution of the 20 amino acid frequencies in different taxonomic groups. Archaea groups (Crenarchaeota, Euryarchaeota, Korarchaeota, Nanoarchaeota and Thaumarchaeota), Bacteria groups (Actinobacteria, Aquificae, Bacteroidetes/Chlorobi, Chlamydiae/Verrucomicrobia, Chloroflexi, Chrysiogenetes, Cyanobacteria, Deferribacteres, Deinococcus-Thermus, Dictyoglomi, Elusimicrobia, Fibrobacteres/Acidobacteria, Firmicutes, Fusobacteria, Gemmatimonadetes, Nitrospirae, Planctomycetes, Proteobacteria, Spirochaetes, Synergistetes, Tenericutes, Thermode sulfobacteria and Thermotogae) and Eukarya groups (Animals, Fungi, Plants and Protists). The taxonomy information was extracted from NCBI classification. The amino acid frequencies in different taxonomic groups were represented as averages plus standard deviations and their expected values based on the universal genetic code were indicated by dash lines.
